# Native envelope-based immunogens derived from critical timepoints in the development of breadth elicit rapid neutralizing antibodies in rabbits

**DOI:** 10.1186/1742-4690-9-S2-P345

**Published:** 2012-09-13

**Authors:** DC Malherbe, AJ Hessell, ND Sather, B Guo, S Pandey, F Pissani, H Robins, S Kalams, L Stamatatos, NL Haigwood

**Affiliations:** 1 Oregon Health and Science University, Beaverton, OR, USA; 2 Seattle BioMed, Seattle, WA, USA; 3 Fred Hutchinson Cancer Research Center, Seattle, WA, USA; 4 Vanderbilt University, Nashville, TN, USA

## Background

HIV-1 evolves rapidly within the host, resulting in the development of diverse variants called a viral “quasispecies” population. A major goal of vaccine efforts is the design of Envelope (Env)-based immunogens effective at eliciting broadly neutralizing antibodies. We hypothesize that B cells become programmed to develop broad NAbs by exposure to Envs presented by the viral quasispecies variants. We propose that similar programming could be achieved by a vaccine concept exposing the host to such Env quasispecies variants isolated from an individual who developed broad NAbs over time.

## Methods

Full-length functional env genes were cloned longitudinally from elite neutralizer CI10014 by single genome amplification, and a combination of in silico sequence analysis and in vitro neutralization was used to select vaccine candidates. Four immunization strategies were tested in rabbits: (1) sequential env evolution as it occurred in CI10014, with multiple clones per timepoint (Sequential); (2) sequential vaccine approach using only one clone per timepoint (Simplified Sequential); (3) an approach uniquely focused on env clones derived from timepoints where env evolution drove the development of breadth (Jump into Breadth); and (4) single env variant (Clonal). The gp160-DNA and gp140-trimer immunogens were co-administered.

## Results

NAbs were detected at six weeks, after only two immunizations and increased after additional immunizations. The Jump into Breadth strategy elicited significantly higher NAbs than the Clonal and Sequential strategies. Modest heterologous neutralization was obtained against Tier 1 clade A and B viruses.

## Conclusion

Exposure to env immunogens derived from timepoints preceding and contemporaneous with the appearance of neutralization breadth elicited higher NAbs than exposure to a single variant or a longitudinal collection of Envs. This study explores the use of multiple native, related HIV-1 Envs as immunogens and emphasizes the critical importance of understanding the development of breadth in an elite neutralizer subject.

